# Homœopathy in Our Colleges

**Published:** 1887-06

**Authors:** 


### HOMŒOPATHY IN OUR COLLEGES.

There is a way of conveying a *false* impression to one's hearers or readers by telling the truth, but not the whole truth. Such an attempt was made in the April issue of the *Medical Advance** in referring to a resolution, introduced at the last meeting of the I. H. A., to indorse the Homœopathic College of Missouri. Such a resolution was introduced and was laid on the table on the motion of Dr. Kent. A little admission the *Advance* overlooked. During the discussion of this resolution the sentiment of the members was clearly shown to be that while the St. Louis College, under Dr. Kent and his pupils, was doing more for pure Homœopathy than any other college, yet there were others in the different colleges who were also doing all in their power for pure Homœopathy. It was thought it would be unjust to these teachers, striving as they were against immense difficulties to do their duty, to indorse one college. Dr. Kent understood this sentiment, as did others present, and was man

---

*"Had you been present at the I. H. A. meeting at Saratoga last June you would have been witness to a similar attempt, *i. e*, booming one college at the expense of all the rest; but it was so palpable a mistake that it was quickly laid on the table, and the participants erased it from the proceedings."—*Medical Advance*, p. 401, April, 1887.

We ask the *Advance* to kindly furnish the profession with the names of those who "erased it from the proceedings." As is implied by the language used, this erasure was done unlawfully and without the consent of the Association. Why did the editors of the *Advance*, then acting as president and secretary of the I. H. A., allow such unlawful erasure?

enough to move to table the resolution. Although this resolution was not passed, every member of the I. H. A. knows of the work Dr. Kent is doing, and *how* he is doing it, and is thankful for it. We all know there is no homœopathic college in this country where Homœopathy is taught as it is at St. Louis. We all also know that there are in several colleges able men who are doing their best to teach, by precept and by example, the Homœopathy of Hahnemann, but, owing to the conditions under which they labor, these physicians cannot do the work Dr. Kent is doing. We desire to detract naught from any man's praise, nor to decry one to bolster up another.

Moreover, the members of the I. H. A. know full well there is scarcely a college in this broad land in which Homœopathy, as Hahnemann taught it, is not ridiculed, misrepresented, and falsely taught by most of those who pretend to teach it! In the college in this city the homœopathic teaching is a farce; one could as well learn Homœopathy at one of the old school colleges as there. Farrington, alas! is dead, and the College is empty of teachers of Homœopathy. A pupil at the Missouri College *can* learn Homœopathy; at most of the other colleges he *can't* learn, for none is taught. In the one instance, eclecticism may be taught by some of the professors; in most of the other colleges eclecticism is taught by all. In the one case, Homœopathy is ably taught by some; in the other instance, Homœopathy is derided and abused by all!

We maintain, as we have written before, it is the duty of every pure Hahnemannian to see that his friends and pupils go to a college where Homœopathy is taught and respected. And we would earnestly recommend all Hahnemannians to support Dr. Kent in every way in his work. Perhaps some day those who are now working in the other colleges may be able to overcome the overwhelming eclectic influences which now surround them and place their institutions upon pure homœopathic principles. We shall be only too glad to do all in our power to help them.

The *Advance* wants to know where there is to be found a college of any kind which is perfect. This, it would seem, appears to be considered an argument against the St. Louis College! If so it is a remarkable argument. No one ever claimed any person or college was perfect. How could such claim be made, seeing *even the Advance* is far from being perfect!

